# Improving the accuracy of genomic prediction for meat quality traits using whole genome sequence data in pigs

**DOI:** 10.1186/s40104-023-00863-y

**Published:** 2023-05-10

**Authors:** Zhanwei Zhuang, Jie Wu, Yibin Qiu, Donglin Ruan, Rongrong Ding, Cineng Xu, Shenping Zhou, Yuling Zhang, Yiyi Liu, Fucai Ma, Jifei Yang, Ying Sun, Enqin Zheng, Ming Yang, Gengyuan Cai, Jie Yang, Zhenfang Wu

**Affiliations:** 1 College of Animal Science and National Engineering Research Center for Breeding Swine Industry, South China Agricultural University, Guangzhou, 510642 China; 2 Guangdong Provincial Key Laboratory of Agro-animal Genomics and Molecular Breeding, Guangzhou, 510642 China; 3 College of Animal Science and Technology, Zhongkai University of Agriculture and Engineering, Guangzhou, 510225 China; 4 Yunfu Subcenter of Guangdong Laboratory for Lingnan Modern Agriculture, Yunfu, 527400 China

**Keywords:** Genomic prediction, Meat quality, Pigs, Whole genome sequence

## Abstract

**Background:**

Pork quality can directly affect customer purchase tendency and meat quality traits have become valuable in modern pork production. However, genetic improvement has been slow due to high phenotyping costs. In this study, whole genome sequence (WGS) data was used to evaluate the prediction accuracy of genomic best linear unbiased prediction (GBLUP) for meat quality in large-scale crossbred commercial pigs.

**Results:**

We produced WGS data (18,695,907 SNPs and 2,106,902 INDELs exceed quality control) from 1,469 sequenced Duroc × (Landrace × Yorkshire) pigs and developed a reference panel for meat quality including meat color score, marbling score, L* (lightness), a* (redness), and b* (yellowness) of genomic prediction. The prediction accuracy was defined as the Pearson correlation coefficient between adjusted phenotypes and genomic estimated breeding values in the validation population. Using different marker density panels derived from WGS data, accuracy differed substantially among meat quality traits, varied from 0.08 to 0.47. Results showed that MultiBLUP outperform GBLUP and yielded accuracy increases ranging from 17.39% to 75%. We optimized the marker density and found medium- and high-density marker panels are beneficial for the estimation of heritability for meat quality. Moreover, we conducted genotype imputation from 50K chip to WGS level in the same population and found average concordance rate to exceed 95% and *r*^2^ = 0.81.

**Conclusions:**

Overall, estimation of heritability for meat quality traits can benefit from the use of WGS data. This study showed the superiority of using WGS data to genetically improve pork quality in genomic prediction.

**Supplementary Information:**

The online version contains supplementary material available at 10.1186/s40104-023-00863-y.

## Background

Duroc × (Landrace × Yorkshire) (DLY) commercial pigs contribute to humans with a substantial fraction of meat supply to meet the consumer’s growing demands for animal protein. Pork meat quality can directly affect customer purchase tendency and consequently attract more attention in the pork production [1]. It is expected that pork quality can be substantially improved with the utilization of genetic approaches [2–4]. Recently, with the development of high-density single nucleotide polymorphisms (SNP) chips for pig genotyping, researchers employed genome-wide association study (GWAS) to detect the quantitative trait loci (QTLs) and genes affecting meat quality in pigs. These findings provide important insights into understanding the underlying genetic basis of meat quality in pigs and had laid the groundwork for facilitating their genetic improvement when using genomic prediction (GP). Genomic prediction is a useful methods that relies on linkage disequilibrium (LD) between SNPs and causative mutations to predict breeding values using markers across the whole genome in animal and plant breeding [5]. It had been implemented to improve meat quality in pigs [6] and Nelore cattle [7]. However, genetic improvement for pork quality has been slow since the phenotyping is cost-expensive, especially in purebred pigs, because purebred nucleus lines are mainly used to produce commercial pigs. Therefore, establishing a large-scale reference population for meat quality traits in crossbred DLY pigs are economically viable and feasible [6]. Thus, it is required to achieve high prediction accuracies of genomic estimated breeding values (GEBV) in GP, which can facilitate the genetic improvement of pork meat quality.

Prediction accuracy of GP in animals has subject to many factors including but not limited to heritability of the analyzed traits, reference sample sizes, and marker density [8, 9]. In pigs, a number of studies of GP used SNP panels (such as 60K, 80K, and imputed 650K SNP array) to estimated GEBVs of complex traits and the prediction accuracies varied among traits [10, 11]. With the development of high throughout DNA sequencing technology, whole genome sequence (WGS) data, which contain genetic variants in high LD with or theoretically covering all causative mutations that are responsible for complex traits, provides an opportunity to increase the prediction accuracy in GP. However, it has been shown that using the complete WGS data did not result in significant improvement in prediction accuracy in comparison with that using SNP chip panels [12–14]. For purebred population, a study in a purebred commercial brown layer chicken line showed that no increase was gained in prediction accuracy when complete WGS data was used in GP scheme compared to using SNP array data [15]. For crossbred population, very little improvement over 50K SNP chip prediction was observed when using all WGS data in a crossbred sheep population [16]. Many factors have an influence on the prediction accuracy with WGS data including the genetic structure of trait and LD [17]. These findings implied that marker density has an essential influence on prediction accuracy in GP. Therefore, it is of important theoretic and practical significance to optimize the marker density in GP scheme. Moreover, another reason for poor prediction accuracy using complete WGS data is that additional insertion-deletion (INDEL) variants that capturing missing heritability for complex traits were not incorporated into prediction models, although it also has high LD with causative mutations [18]. Recent results has shown that prediction accuracies were increased using INDEL compared with that using SNPs [18].

The aim of this study was to (i) evaluate the prediction accuracy of genomic best linear unbiased prediction (GBLUP) and MultiBLUP [19] for meat quality traits (including meat color score, marbling score, L*: lightness, a*: redness, and b*: yellowness) in 1,469 crossbred commercial DLY pigs using WGS data and optimized the marker density in GP scheme; (ii) capture missing heritability of meat quality traits using INDEL variants from WGS data. Genotype imputation, leveraging LD to infer genotypes at undetected polymorphic loci [20], has been proven to a cost-effective approach that greatly increase the density of genotypes to WGS level data and is widely used in the genetic studies. We therefore performed genotype imputation in the same population to acquire the accuracy of imputation from commercial medium-density marker panel (50K) to WGS level data.

## Material and methods

### Ethics statement

The procedure for collecting tissue samples from pigs was performed with the approval of the ethics committee of South China Agricultural University (Guangzhou, China) under 2018F098.

### Experiment animals, sample collection and phenotyping

In the current study, 1,469 crossbred commercial DLY pigs (born from 2018 to 2019) that raised on four farms of Wen’s Foodstuffs Group Co., Ltd. (Guangdong, China) were used to collect phenotypes and genotypes as described previously [21]. Briefly, a total of 84 Duroc boars of U.S. origin (S21), Canadian origin (S22), and Taiwan province of China origin (S23) were mated to 397 Landrace × Yorkshire sows to produce offspring. Then, the piglets were moved to the fattening pen. All 1,469 animals were slaughtered for phenotype recording at an average body weight of 115 kg in 13 batches. Firstly, in order to collect phenotypic values of pork quality, *longissimus thoracis* (LT) muscle was removed from the left side of each carcass and samples of LT were kept at 4 ℃ inside the refrigerator until the marbling score, meat color score, L*, a*, and b* were measured at 12 h post mortem. Marbling score and meat color traits (meat color score, L*, a*, and b*) measurements were performed on LT muscle as described previously [22]. Briefly, three meat color parameters (L*, a*, and b*) were measured on the exposed cut surface after blooming for 30 min of the LT at 12 h post mortem using a CM-2600d/2500d Minolta Chromameter (Tokyo, Japan) with an 8-mm measuring port, D65 illuminant, and one trained observer. Meat color score (MC, from 1 to 6; pale to dark) and marbling score (from 1 to 10; devoid to overly abundant), which also refers to intramuscular fat content (IMF), were rated by one trained personnel on the same cut surface of LT muscle following the U.S. National Pork Producer Council guidelines [23].

A single-trait animal model was used to calculate corrected phenotypic values (*Yc*) for meat quality traits using PREDICTF90 module in BLUPF90 software [24]. Fixed effects included farm (level = 4), sex (level = 2), and slaughter batch (level = 13). Then, the corrected phenotypic value *Yc* was used for subsequent analyses.

### Whole-genome sequencing, variant detection and filtering

For the 1,469 DLY pigs, each individual was sequenced (~10 × coverage) on Illumina Hiseq platforms provided by the Novogene Biotech Co., Ltd. (Beijing, China) with 150 bp paired-end reads. Firstly, the FASTQ format sequence reads were aligned to the pig reference genome assembly *Sscrofa*11.1 via BWA-MEM-0.7.12 [25] with default settings. Secondly, the mapping results were sorted using SAMtools 1.9 [26]. Then, the Sentieon DNAseq pipeline (version 202010) [27] were used to conduct variant calling following the “best practices” algorithms of GATK (Genome Analysis Toolkit), and followed functions (mainly used) from Sentieon were applied: “--algo LocusCollector” and “--algo Realigner” functions were applied to remove duplicate reads and realigned indels; “--algo QualCal” function was invoked to conduct base quality score recalibration (BQSR); “--algo Haplotyper” function was used to call variants in GVCF format; “--algo GVCFtyper” function was used to conduct joint calling by combining GVCFs across all samples and consequently, a population VCF was generated. Further, GATK v4.0.2.1 [28] with “VariantFiltration” module was used to detect SNPs using following criteria: “QD < 2.0, FS > 60.0, SOR > 3.0, MQ < 40.0, MQRankSum < −12.5, ReadPosRankSum < −8.0”; For INDEL excluding, we used the parameters “QD < 2.0, QUAL < 50.0, FS > 100.0, and ReadPosRankSum < −20.0”, as recommended by GATK’s best practices. After filtering, 27,777,985 autosomal SNPs and 5,341,470 autosomal INDELs were detected. Finally, 21,708,028 SNPs and 3,000,017 INDELs were remained for subsequent analyses with call rate higher than 0.9 and minor allele frequencies (MAF) higher than 0.01 using PLINK 1.9 [29].

### Chip-variants genotyping and genotype imputation

It is expected to use imputed-based WGS data to improve the power and prediction accuracy of GP for many studies that lack of WGS level data. To this end, we performed genotype imputation using a public web server (swimgeno.org) and estimated the accuracy of imputation from commercial medium-density marker panel (50K) to WGS level data [30]. Firstly, the 1,469 DLY pigs were genotyped using the Geneseek GGP Porcine 50 K SNP chip (Neogen, Lincoln, NE, USA). Next, the genotype dataset was converted to *Sscrofa*11.1 after genotyping. Then, the PLINK ped/map formatted SNP array genotypes were submitted to SWIM web server (swimgeno.org) to conduct genotype imputation procedure. By doing genotype imputation, 30,489,782 autosomal SNPs and 4,125,579 autosomal INDELs were acquired. Quality control (QC) were conducted using PLINK 1.9 [29] following the criteria with call rate higher than 0.9 and minor allele frequencies (MAF) higher than 0.01, and results in final 19,348,898 SNPs and 2,725,851 INDELs remaining for subsequent analyses.

### Estimation of genotype imputation accuracy

To validate the performance of pig haplotype reference panel (termed SWIM), we randomly selected 294 (20% of the sequenced 1,469) sequenced pigs to calculate its imputation accuracy for DLY pigs. Herein, we defined the imputation accuracy using two values including the overall concordance rate (CR) between imputed and observed genotypes, which refers to the percentage of genotypes imputed correctly divided by total imputed genotypes, and the squared Pearson correlation coefficient (*r*^2^) between imputed and observed genotypes. We measured CR and *r*^2^ on a per SNP basis and averaged them over SNPs in MAF bins (0.01 in size) or across the whole genome.

### Phylogenetic and population structure analyses

Genetic distances between individuals of the 1,469 DLY pigs were calculated using an identity-by-state (IBS) similarity kinship matrix by PLINK 1.9. In an aim to analyze the population structure of the DLY pigs in this study, we pruned SNPs with LD using PLINK 1.9 with the parameters “-indep-pairwise 50 10 0.6” (*r*^2^ < 0.6) to remain 3,216,966 variants. Principal component analysis (PCA) was conducted on the pruned SNPs dataset using GCTA 1.93.2 [31] for 1,469 pigs. Linkage disequilibrium was calculated via PopLDdecay software [32] on animals within the S21, S22, and S23 Duroc boars-produced populations with SNPs of MAF > 0.05. Additionally, we calculated the Euclidean distance between individuals using “distances” package in R.

### Pre-selection of SNPs and INDELs

From the 21,708,028 SNPs and 3,000,017 INDELs of WGS data and the 19,348,898 SNPs and 2,725,851 INDELs of imputed-based WGS data after QC, respectively, common SNPs (18,695,907) and INDELs (2,106,902) that were present on the two sequence datasets were selected as final subsets of WGS data and were used for subsequent analyses. These different variant panels also can be used to access the prediction accuracy of GP when using variants from imputed-based WGS data. In an attempt to optimize the marker density of variants in GP scheme and compare the prediction accuracy when different variant types (SNP and INDEL) were incorporated into model, we constructed three levels density of marker subsets, including low-density variant panels (1K, 3K, 10K, 30K for SNPs and INDELs), medium-density variant panels (100K, 500K for SNPs and INDELs), and high-density variant panels (1,000K, 5,000K, 10,000K for SNPs; 1,000K for INDELs) using PLINK 1.9 with parameter “--thin” (e.g., --thin 0.0005 means to keep only a random 0.05% of variants, modify this parameter to get panels with different number of variants). Accordingly, we make a combination of the SNPs and INDELs in the sequenced datasets selected above with the same marker density into one model. Besides, it has been shown that the degree of LD of WGS data had an effect on prediction accuracy of GP [17]. We subsequent pruned SNP datasets with LD (*r*^2^ < 0.2, 0.3, 0.6, 0.8) using common WGS data to construct LD-based SNPs panels. Finally, a total of 28 panels (the complete WGS SNP data was included) that represent different marker density, categories of variants were generated. The GBLUP and MultiBLUP models were used for genomic prediction to estimate the GEBV of meat quality traits with these different variant panels.

### Estimation of heritability

The proportion of phenotypic variance explained by preselected SNP and INDEL panels with different marker density were calculated using GCTA 1.93.2 software with “GREML” function [31]. The pre-adjusted phenotypes *Yc* were used as response variable in the model. For partitioning contributions to heritability by different types and different marker densities of variants, we estimated the genetic relationship matrix (GRM) between pairs of animals from a set of SNPs and INDELs, respectively. In this step, we set the parameter with “--make-grm-alg 1” to calculate GRM using the equation as describe in [9]. Then, REML (restricted maximum likelihood) analysis was performed to calculate the variance explained by the variant datasets.

### Genomic prediction models

The GEBV was calculated using GBLUP model described as follows [9]:

$$\boldsymbol{y}=\boldsymbol{1}{\mu}+{\varvec{Z}}\boldsymbol{g}+\boldsymbol{e},$$

where ***y*** is an *N* × 1 vector of *Yc*, **1** is the *N* × 1 vector of ones, $\mu$ is the overall means, ${\varvec{Z}}$ refers to a design matrix relating phenotypes to the additive genetic values, $\boldsymbol{g}$ is the vector of the genomic values captured by the genetic variants (SNP or INDEL), following a normal distribution of $\boldsymbol{g}$ ~ *N*(0, ${\varvec{G}}{\sigma }_{g}^{2}$), where ${\sigma }_{g}^{2}$ is the additive genetic variance and ${\varvec{G}}$ is the genomic relationship matrix derived by SNPs or INDELs; $\boldsymbol{e}$ is the vector of random residual with $\boldsymbol{e}$ ~ *N*(0, ${\varvec{I}}{\sigma }_{e}^{2}$), where ${\sigma }_{e}^{2}$ is the random residual variance and ${\varvec{I}}$ is an identity matrix.

The MultiBLUP model including two random genetic effects was described as follows [19]:

$$\boldsymbol{y}=\boldsymbol{1}{\mu}+{{\varvec{Z}}}_{{\varvec{f}}}\boldsymbol{g}_{f}+{{\varvec{Z}}}_{{\varvec{r}}}\boldsymbol{g}_{r}+\boldsymbol{e},$$

where ***y***, **1**, $\mu$ and $\varvec {e}$ are same as GBLUP model, $\boldsymbol{g}_{f}$ and $\boldsymbol{g}_{r}$ are the vectors of the genomic values captured by the genetic variants INDELs and SNPs, respectively. $\boldsymbol{g}_{f}$ follows a normal distribution of $\boldsymbol{g}_{f}$~*N*(0, ${{\varvec{G}}}_{{{\varvec{g}}}_{{\varvec{f}}}}{\sigma }_{gf}^{2}$), where ${\sigma }_{gf}^{2}$ is the additive genetic variance and ${{\varvec{G}}}_{{{\varvec{g}}}_{{\varvec{f}}}}$ is the genomic relationship matrix derived by INDELs; $\boldsymbol{g}_{r}$ follows a normal distribution of $\boldsymbol{g}_{r}$~ *N*(0, ${{\varvec{G}}}_{{{\varvec{g}}}_{{\varvec{r}}}}{\sigma }_{gr}^{2}$), where ${\sigma }_{gr}^{2}$ is the additive genetic variance and ${{\varvec{G}}}_{{{\varvec{g}}}_{{\varvec{r}}}}$ is the genomic relationship matrix derived by SNPs; ${{\varvec{Z}}}_{{\varvec{f}}}$ and ${{\varvec{Z}}}_{{\varvec{r}}}$ refer to design matrices relating phenotypes to the additive genetic values. The ${\varvec{G}}$**, **${{\varvec{G}}}_{{{\varvec{g}}}_{{\varvec{f}}}}$**,** and ${{\varvec{G}}}_{{{\varvec{g}}}_{{\varvec{r}}}}$ matrices were calculated as follows [9]:

$${\varvec{G}}= \frac{{{\varvec{M}}{\varvec{D}}{\varvec{M}}}^{T}}{2{\sum }_{i=1}^{m}{p}_{i}(1-{p}_{i})},$$

where ${\varvec{M}}$ is the matrix of genotypes from sequenced data, ${\varvec{D}}$ is the identify matrix (the same to ${\varvec{I}}$), $m$ is the number of markers in the panel and ${p}_{i}$ is the MAF of $i^{\mathrm{th}}$ marker (SNP or INDEL).

### Cross-validation and prediction bias

In this study, prediction accuracy was defined as the Pearson correlation coefficient between *Yc* and GEBV in the validation population, and was obtained by a five-fold cross-validation (CV) with five repetitions. Briefly, for each repetition, the 1,469 individuals were randomly split into five subgroups. In each round of five-fold CV, the five subgroups were successively treated as a validation population, and the remaining four subgroups were treated as the reference population. The slope of the regression of *Yc* on GEBV for animals in the validation population was calculate to measure the degree of inflation or deflation of GP. Average prediction accuracy and the bias values for the 25 CV round per trait were reported.

## Results

### Population structure and relationship between kinship and phenotypes among pig reference populations

**Fig. 1:**
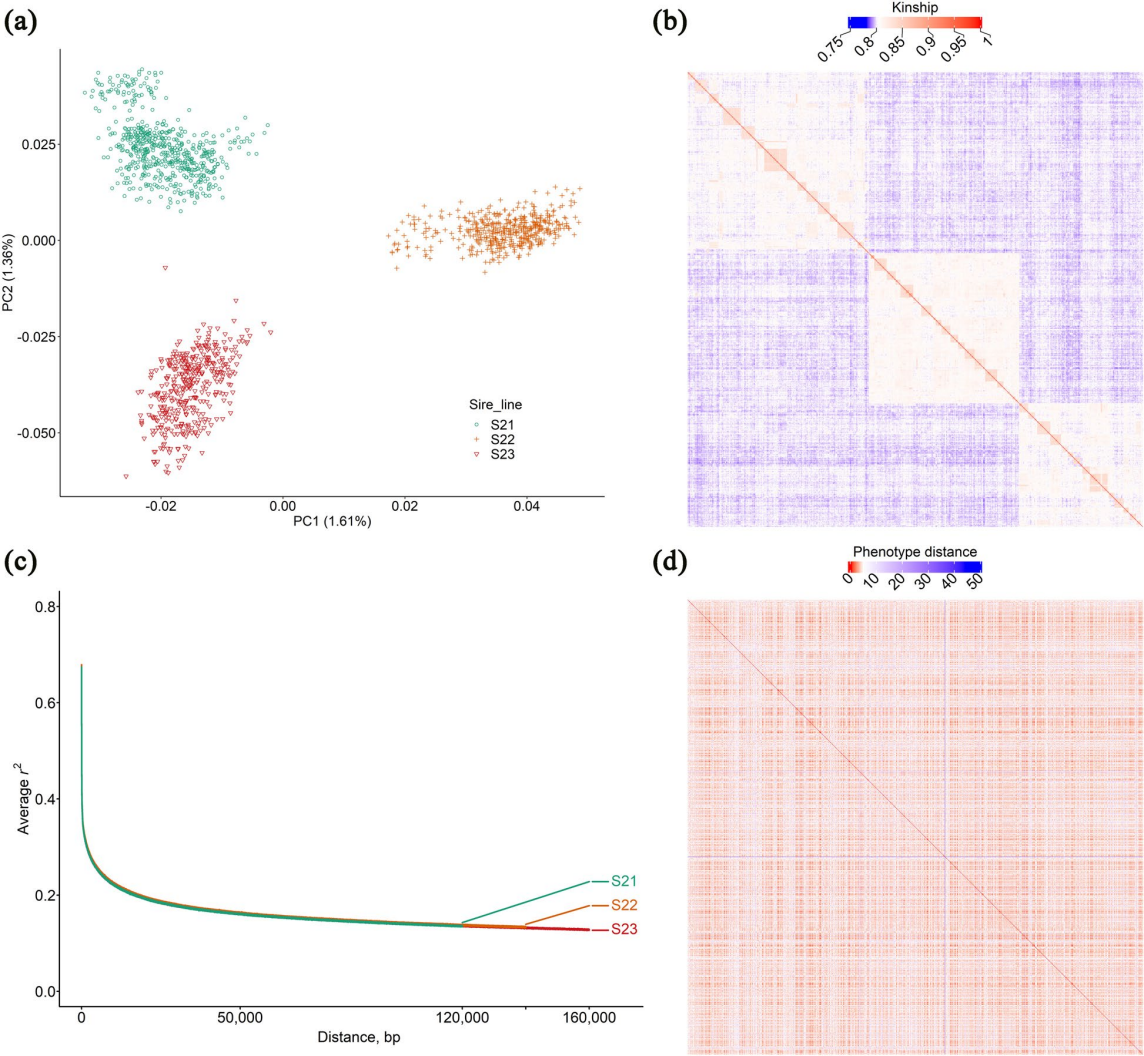
Genetic structure of the DLY pig populations. **a** Scatter plot of the first two principal components of genotypes matrix for common (MAF > 0.01) and LD-pruned SNPs. **b** Genetic distances between individuals of the 1,469 DLY pigs that calculated using an identity-by-state similarity kinship matrix by PLINK v1.9. The individuals are sorted based on pedigree information to make littermates in tandemly order. **c** Pair-wise LD in three DLY subpopulations. Linkage disequilibrium was calculated via PopLDdecay software on animals within the S21, S22, and S23 subpopulations with SNPs of MAF > 0.05. **d** The relationship between individuals based on phenotype distance

To better elucidate the population structure and genetic distance among the studied populations, we showed the two-dimensional principal components plot of the three DLY subpopulations and the relationship between individuals for all pairs. As shown in Fig. 1a, the three lines were separated into distinct clusters, the first principal component of the genotypes separated the S21, S23 boar Duroc lines and S22 boar Duroc line, and the second principal component of the genotypes separated the S21 boar Duroc line and S23 boar Duroc line. Similar pattern of genetic diversity was observed from IBS matrix, and the genetic distance values ranged from 0.75 to 1 (Fig. 1b). The individuals are sorted based on pedigree information to make littermates in tandemly order. However, LD between variants in the three subpopulations was extensive but has similar LD decay trend among lines (Fig. 1c). Furthermore, the relationship between individuals based on phenotype distance also shown the three DLY pig subpopulations did not have high phenotypic distance (Fig. 1d). Summary statistics of meat quality traits in pigs are listed in Additional file 1: Table S1. The relationship between the Euclidean distance calculated with IBS matrix and phenotype values shown that genetic data and phenotypes was slightly anticorrelated, but the degree was minor (Additional file 2: Fig. S1).

### Accuracy of genotype imputation

**Fig. 2:**
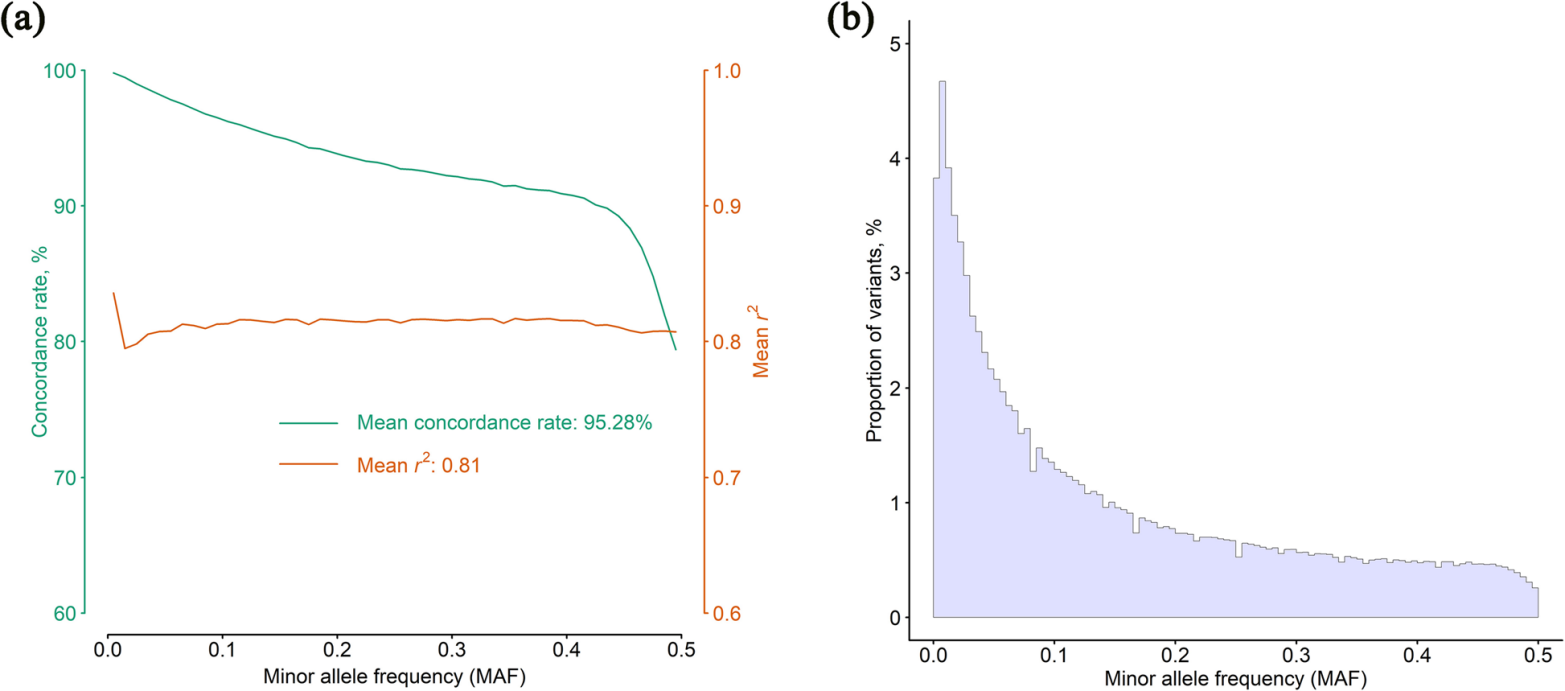
Accuracy of genotype imputation over MAF and distribution of allele frequency for all variants from WGS. **a** Concordance rate and *r*^2^ of imputed versus observed genotypes. **b** Distribution of MAF for all variants in the reference panel

A total of 294 pigs were extracted as target population to evaluate the imputation accuracy. We achieved an average CR across all variants in excess of 95.28% and *r*^2^ of 0.81, as shown in Fig. 2a. These results suggested that the imputed-based WGS data have high genotype imputation precision and is sufficient for genetic analyses. Moreover, the average CR is sensitive to MAF as the curve goes down faster with MAF higher than 0.40. For the variants with a MAF lower than 0.40, the CR was greater than 90%. Accordingly, proportion of variants over distribution of MAF for all variants in the target population also suggested that the count of variants with MAF higher than 0.4 was small (Fig. 2b). Generally, the accuracy of genotype imputation increased along with the number of markers in MAF bins increased (Fig. 2b).

### Heritability captured by different categories of variants

In the current study, common SNPs (18,695,907) and INDELs (2,106,902) that were present on the WGS and imputed-based WGS datasets were selected as final subsets of variants datasets for subsequent genomics analyses. The number of variants of the totally 28 panels that represent different marker density, categories of variants are listed in Table 1 and Additional file 3: Table S2. The estimated heritability of the meat quality traits captured by SNPs and INDELs with different marker densities are listed in Table 1. In Table 1, SNP+INDEL panels refer to variants that used in MultiBLUP model. The estimated heritability for meat quality traits using LD pruned SNPs are listed in Additional file 4: Table S3. Briefly, the estimated heritability of the meat quality traits did not have substantial difference within low-density, medium-density and high-density SNP panels and INDEL panels, but the INDEL panels explained higher phenotypic variance of meat quality than that explained by SNP panels in some cases, implying that INDELs can captured missing heritability. However, the estimated heritability of the meat quality traits has substantial difference between low-density and medium-density, high-density SNP panels and INDEL panels. We observed substantial increase in heritability when marker density was increased to at least 100K level (by an increase of 100% ~ 175%). For example, the heritability estimated by low-density variants panels for IMF was 0.13 (1K), however, the heritability estimated by medium and high-density variants panels was 0.26. The heritability estimated by low-density variants panels for a* was 0.28 (1K), however, the value was 0.61 estimated by medium and high-density variants panels. In addition, we observed no increase in heritability when the marker density of WGS data was reached to more than 5 million (5,000K). These results demonstrated that medium and high-density marker panels and variant category are beneficial for the estimation of heritability for meat quality traits.

**Table 1:** The number of preselected SNPs and INDELs from common variants dataset that were present on the WGS and imputed-based WGS and estimated heritability

Traits^a^	Variants' number and heritability^b^	Variants type^c^	Marker density								
			1K	3K	10K	30K	100K	500K	1,000K	5,000K	10,000k
	Number of preselection variants	SNP	1,017	2,968	10,009	29,931	100,070	500,006	1,000,006	4,999,686	10,001,734
		INDEL	1,018	3,063	9,973	30,122	100,130	500,971	1,000,565		
IMF	Estimated heritability (Mean ± SE)	SNP	0.13 ± 0.03	0.18 ± 0.04	0.23 ± 0.04	0.14 ± 0.05	0.25 ± 0.05	0.26 ± 0.05	0.26 ± 0.05	0.26 ± 0.05	0.26 ± 0.05
		INDEL	0.13 ± 0.03	0.18 ± 0.03	0.24 ± 0.04	0.25 ± 0.05	0.25 ± 0.05	0.26 ± 0.05	0.27 ± 0.05		
		SNP+INDEL	0.16 ± 0.05	0.20 ± 0.04	0.25 ± 0.05	0.25 ± 0.05	0.26 ± 0.05	0.26 ± 0.05	0.27 ± 0.05		
MC		SNP	0.15 ± 0.03	0.20 ± 0.04	0.29 ± 0.05	0.28 ± 0.05	0.28 ± 0.05	0.30 ± 0.05	0.30 ± 0.05	0.30 ± 0.05	0.30 ± 0.05
		INDEL	0.12 ± 0.03	0.21 ± 0.04	0.24 ± 0.04	0.29 ± 0.05	0.29 ± 0.05	0.30 ± 0.05	0.30 ± 0.05		
		SNP+INDEL	0.17 ± 0.03	0.24 ± 0.04	0.28 ± 0.05	0.29 ± 0.05	0.29 ± 0.05	0.30 ± 0.05	0.30 ± 0.05		
L*		SNP	0.07 ± 0.03	0.12 ± 0.03	0.15 ± 0.04	0.14 ± 0.04	0.14 ± 0.04	0.15 ± 0.04	0.15 ± 0.04	0.15 ± 0.04	0.15 ± 0.04
		INDEL	0.07 ± 0.02	0.12 ± 0.03	0.13 ± 0.04	0.14 ± 0.04	0.15 ± 0.04	0.15 ± 0.04	0.15 ± 0.04		
		SNP+INDEL	0.08 ± 0.03	0.14 ± 0.04	0.15 ± 0.04	0.15 ± 0.04	0.15 ± 0.04	0.15 ± 0.04	0.15 ± 0.04		
a*		SNP	0.28 ± 0.03	0.34 ± 0.04	0.51 ± 0.05	0.56 ± 0.05	0.59 ± 0.05	0.61 ± 0.05	0.61 ± 0.05	0.61 ± 0.05	0.61 ± 0.05
		INDEL	0.27 ± 0.03	0.39 ± 0.04	0.47 ± 0.05	0.54 ± 0.05	0.60 ± 0.05	0.62 ± 0.05	0.62 ± 0.05		
		SNP+INDEL	0.36 ± 0.04	0.44 ± 0.04	0.55 ± 0.05	0.58 ± 0.05	0.60 ± 0.05	0.62 ± 0.05	0.62 ± 0.05		
b*		SNP	0.04 ± 0.02	0.08 ± 0.03	0.11 ± 0.04	0.11 ± 0.04	0.11 ± 0.04	0.11 ± 0.04	0.11 ± 0.04	0.11 ± 0.04	0.11 ± 0.04
		INDEL	0.05 ± 0.02	0.09 ± 0.03	0.09 ± 0.04	0.11 ± 0.04	0.12 ± 0.04	0.11 ± 0.04	0.12 ± 0.04		
		SNP+INDEL	0.06 ± 0.03	0.10 ± 0.04	0.11 ± 0.04	0.11 ± 0.04	0.12 ± 0.04	0.12 ± 0.04	0.12 ± 0.04		

^a^*IMF* Intramuscular fat content (also refers to marbling score in this study), *MC* Meat color, L* Lightness, a* Redness, b* Yellowness

^b^*SE* Standard error

^c^SNP+INDEL: variants used in MultiBLUP

### Genomic prediction accuracy of GBLUP and MultiBLUP of meat quality traits

**Fig. 3:**
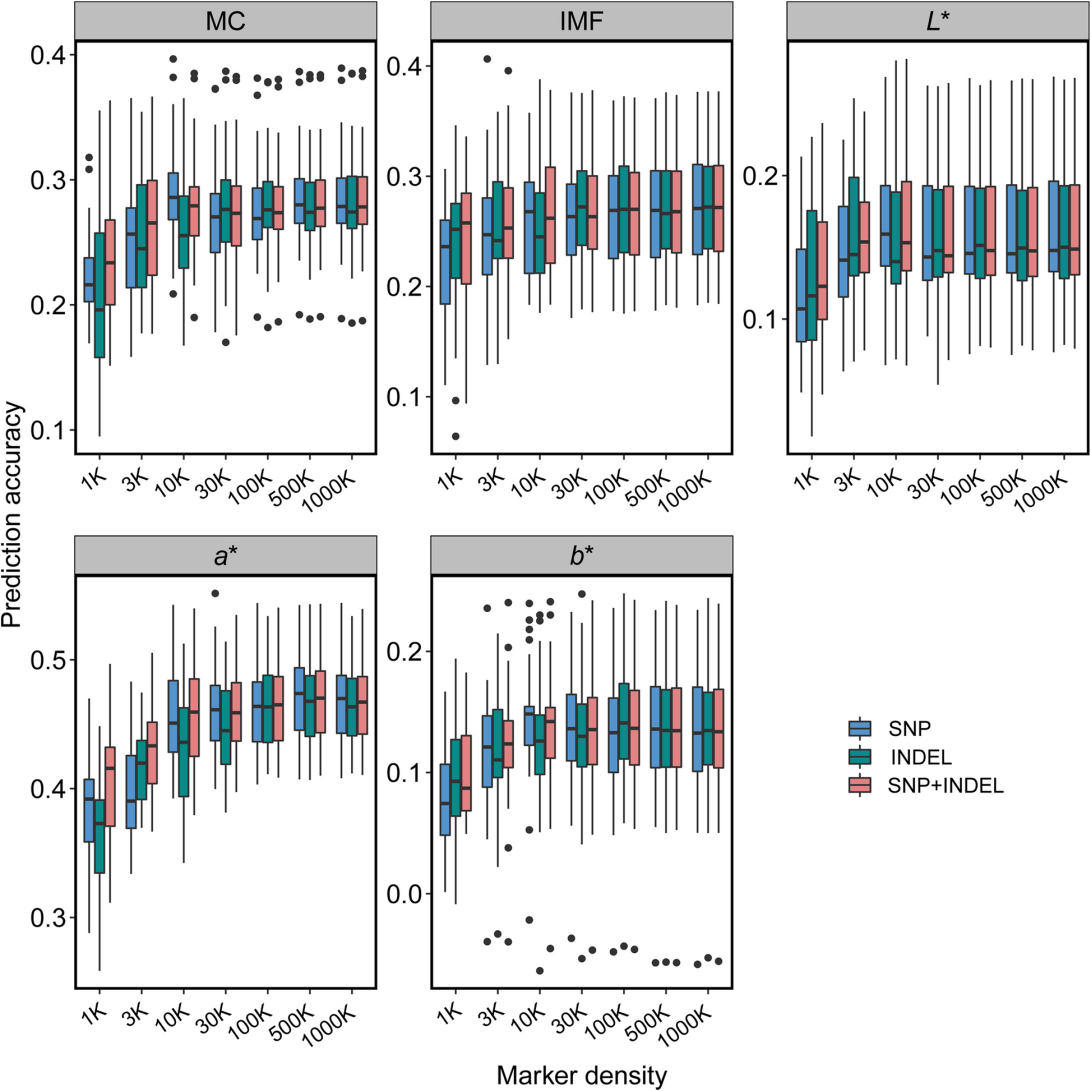
Box plots of genomic prediction accuracy over marker density for meat quality traits. The prediction accuracy was defined as the Pearson correlation coefficient between adjusted phenotypes and GEBV in the validation population, and was obtained by a five-fold cross-validation with five repetitions. “SNP” represents the accuracy of GBLUP using different SNP panels. “INDEL” represents the accuracy of GBLUP using different INDEL panels. “SNP+INDEL” refers to the accuracy of MultiBLUP using INDEL as a random effect

**Fig. 4:**
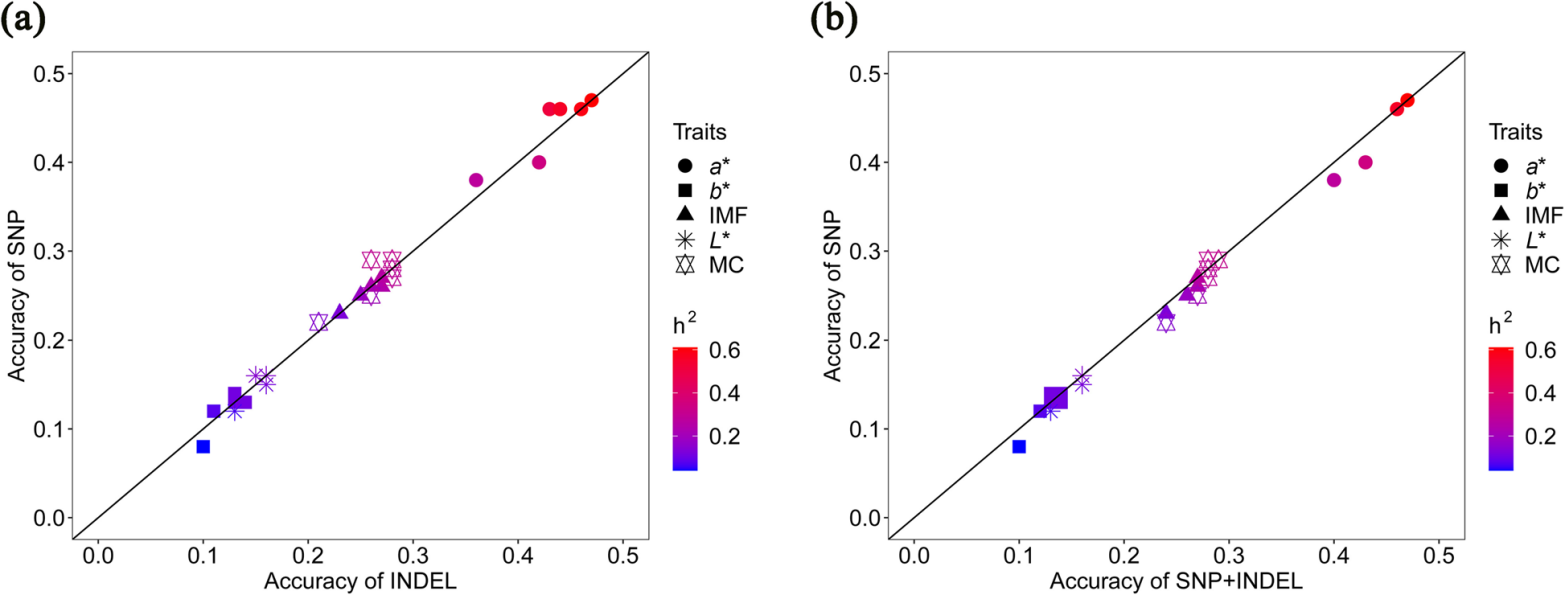
Average accuracy of genomic prediction using GBLUP and MultiBLUP for meat quality traits. Points showed the average accuracy of each five-fold cross-validation with five repetitions. **a** The average accuracy of GBLUP using different SNP panels versus average accuracy of GBLUP using different INDEL panels. **b** The average accuracy of GBLUP using different SNP panels versus average accuracy of MultiBLUP using different INDEL panels as random effects

Box plots of the accuracy of five-fold cross-validation with five repetitions against variants numbers for meat quality traits are shown in Fig. 3. The results of same marker densities between SNPs and INDELs are shown. The mean accuracy and bias values of both GBLUP and MultiBLUP using SNPs and INDELs is shown in Table 2 and Fig. 4a–b. For IMF trait, the results showed that the accuracy of GBLUP using low-density SNP panels and INDEL panels ranged from 0.23 to 0.26, 0.23 to 0.27, respectively. The accuracy of GBLUP using medium-density and high-density SNP panels and INDEL panels was same and reached to 0.27. Compared with the accuracy of GBLUP using 1K variant panels, the best accuracy values of GBLUP and MultiBLUP with 1,000K SNPs (INDELs) were represented by increases of 17.39% in IMF. For the MC trait, the results showed that the accuracy of GBLUP using low-density SNP panels and INDEL panels ranged from 0.22 to 0.27 and 0.21 to 0.28, respectively. The values of accuracy of GBLUP using medium-density and high-density SNP panels (INDEL panels) ranged from 0.28 to 0.29 (INDEL panels: 0.28). By using SNPs with high-density panels, the best accuracy values were represented by increases of 31.82% for GBLUP and MultiBLUP, compared to GBLUP with 1K panel. For L* trait, GBLUP using low-density SNP panels and INDEL panels resulted in low prediction accuracy, ranged from 0.12 to 0.16 and 0.13 to 0.16, respectively. The results of accuracy of GBLUP using medium-density and high-density SNP panels and INDEL panels were same, reached to 0.16. By using SNPs with high-density panels, the best accuracy values were represented by increases of 33.33% for GBLUP and MultiBLUP, compared to GBLUP with 1K panel. For a* trait, the results showed that the accuracy of GBLUP using low-density SNP panels and INDEL panels ranged from 0.38 to 0.46 and 0.36 to 0.44, respectively. The values of accuracy of GBLUP using medium-density and high-density SNP panels and INDEL both ranged from 0.46 to 0.47. Compared with the accuracy of GBLUP using 1K variant panels, the best accuracy values of GBLUP and MultiBLUP with 1,000K SNPs (INDELs) were represented by increases of 23.68% in a*. For b* trait, the best accuracy value was 0.14 for GBLUP using 30K (10K) SNP panel and 100K (1,000K) INDEL panel and for MultiBLUP using low-density panels (except for 1K panel). Compared to GBLUP with 1K SNP panel, the best accuracy value of GBLUP and MultiBLUP were represented by increases of 75% in b* trait. In general, compared with the GBLUP using complete WGS data, we observed no increases in accuracy for all meat quality traits, except for the b* trait, when the density of WGS data was reached to more than 500,000 (500K). The results of accuracy of GBLUP and MultiBLUP were increased with the increase of heritability of meat quality traits (Fig. 4a and b). Notably, it is surprising that the GEBV accuracy of MultiBLUP model outperform GBLUP model for all the analyzed meat quality traits when using low-density variant panels (less than 30,000 markers) (Fig. 4b).

**Table 2:** The mean accuracy and bias values of genomic prediction using preselected SNPs and INDELs from common variants dataset that were present on the WGS and imputed-based WGS

**Traits**^**a**^	**Model**^**b**^	**Variants type**	**Marker density**								
			**1K**	**3K**	**10K**	**30K**	**100K**	**500K**	**1,000K**	**5,000K**	**10,000K**
IMF	GBLUP	SNP	0.23 ± 1.04	0.25 ± 1.05	0.26 ± 1.01	0.26 ± 1.00	0.27 ± 1.02	0.27 ± 1.02	0.27 ± 1.02	0.27 ± 1.02	0.27 ± 1.01
		INDEL	0.23 ± 1.05	0.25 ± 1.06	0.26 ± 1.02	0.27 ± 1.02	0.27 ± 1.03	0.27 ± 1.02	0.27 ± 1.02		
	MultiBLUP	SNP+INDEL	0.24 ± 1.03	0.26 ± 1.06	0.27 ± 1.02	0.27 ± 1.01	0.27 ± 1.02	0.27 ± 1.02	0.27 ± 1.02		
MC	GBLUP	SNP	0.22 ± 1.03	0.25 ± 1.05	0.29 ± 1.05	0.27 ± 1.05	0.28 ± 1.05	0.29 ± 1.04	0.29 ± 1.04	0.29 ± 1.04	0.29 ± 1.04
		INDEL	0.21 ± 1.11	0.26 ± 1.04	0.26 ± 1.05	0.28 ± 1.04	0.28 ± 1.06	0.28 ± 1.05	0.28 ± 1.05		
	MultiBLUP	SNP+INDEL	0.24 ± 1.06	0.27 ± 1.05	0.28 ± 1.05	0.28 ± 1.04	0.28 ± 1.05	0.29 ± 1.05	0.29 ± 1.05		
L*	GBLUP	SNP	0.12 ± 1.16	0.15 ± 1.04	0.16 ± 1.04	0.16 ± 1.07	0.16 ± 1.06	0.16 ± 1.05	0.16 ± 1.05	0.16 ± 1.05	0.16 ± 1.05
		INDEL	0.13 ± 1.15	0.16 ± 1.08	0.15 ± 1.09	0.16 ± 1.05	0.16 ± 1.05	0.16 ± 1.06	0.16 ± 1.06		
	MultiBLUP	SNP+INDEL	0.13 ± 1.13	0.16 ± 1.05	0.16 ± 1.06	0.16 ± 1.06	0.16 ± 1.05	0.16 ± 1.05	0.16 ± 1.05		
a*	GBLUP	SNP	0.38 ± 1.03	0.40 ± 1.00	0.46 ± 1.01	0.46 ± 1.00	0.46 ± 1.00	0.47 ± 1.00	0.47 ± 1.00	0.47 ± 1.00	0.47 ± 1.00
		INDEL	0.36 ± 0.99	0.42 ± 1.00	0.43 ± 1.00	0.44 ± 1.00	0.46 ± 1.01	0.47 ± 1.01	0.47 ± 1.01		
	MultiBLUP	SNP+INDEL	0.40 ± 1.00	0.43 ± 1.00	0.46 ± 1.01	0.46 ± 1.00	0.46 ± 1.01	0.47 ± 1.01	0.47 ± 1.01		
b*	GBLUP	SNP	0.08 ± 1.34	0.12 ± 1.14	0.14 ± 1.16	0.14 ± 1.15	0.13 ± 1.18	0.13 ± 1.16	0.13 ± 1.16	0.13 ± 1.15	0.13 ± 1.15
		INDEL	0.10 ± 1.35	0.11 ± 1.22	0.13 ± 1.13	0.13 ± 1.18	0.14 ± 1.15	0.13 ± 1.17	0.14 ± 1.16		
	MultiBLUP	SNP+INDEL	0.10 ± 1.31	0.12 ± 1.14	0.14 ± 1.15	0.14 ± 1.15	0.14 ± 1.14	0.13 ± 1.14	0.13 ± 1.14		

^a^*IMF* Intramuscular fat content (also refers to marbling score in this study), *MC* Meat color, L* Lightness, a* Redness, b* Yellowness

^b^*GBLUP* Genomic best linear unbiased prediction, *MultiBLUP* An extended BLUP model that included multiple random effects

### Accuracy from different marker densities with LD-pruned

In the current study, the impact of LD-based marker pruning of WGS data on prediction accuracy for meat quality traits were investigated using GBLUP model. Four different *r*^2^ cutoffs of LD were set to prune SNPs of WGS data. The results of prediction accuracy of GBLUP for meat quality traits with different LD-based SNPs pruning are shown in Additional file 3: Table S2. For IMF, a*, and b*, the accuracy of LD pruned SNPs reached the highest accuracy values to 0.27, 0.47, and 0.13, respectively, compared to GBLUP with the complete WGS data. For L* trait, the prediction accuracy of LD pruned SNPs (*r*^2^ < 0.6, 0.3, and 0.2) were increased by 6.25%, compared with GBLUP using complete WGS data. In contrast, the prediction accuracy of LD pruned SNPs (*r*^2^ < 0.6 and 0.8) decreased in comparison with that using complete WGS data for MC trait. For all meat quality traits, the prediction accuracy of LD pruned SNPs (*r*^2^ < 0.3 and 0.2) could reach the highest level of prediction ability of GBLUP, implying that LD pruned SNPs can substantially improve computing efficiency in constructing G matrix, as the similar pattern as we observed in studying the impact of marker density on accuracy of genomic prediction.

## Discussion

### Impact of genetic structure of reference population, marker density and LD on genomic prediction and heritability estimation for meat quality

Improving the meat quality is of importance for satisfying the needs of high-quality products of consumers and consequently affecting the purchase decision [7]. Despite the promise of improving meat quality using GP, significant challenges have remained to overcome. Meat quality phenotypes usually are measured post-mortem, owing to high phenotyping costs of meat quality and the difficulty in obtaining a large-scale pig WGS dataset, genetic improvement had been slow using genetic strategy especially for GP. This limitation led to insufficient integrating of genomic information, although meat quality should be an essential element in the breeding programs. To address this issue, we herein constructed a large-scale pig reference population for meat quality traits (including marbling score, meat color score, L*, a*, and b*) consisting of 1,469 whole genome sequenced pigs to implement GP. The genetic structure of this reference population consisted of three admixed genomic relationship subpopulations, however, population structure did not have substantial influence on meat quality traits (Fig. 1d), since the three subpopulations have similar LD decay trend (Fig. 1c). Results of previous studies have shown that the genomic prediction accuracy may be limited when using a small population of purebred, but incorporating genomic data from different breeds or populations might result in higher prediction accuracy [33–35]. GP scheme benefit a lot from incorporating data from multiple genomic relationship closely related subpopulations into large-scale reference population [35]. Moreover, using crossbreed commercial DLY pigs to construct reference population in terms of improving meat quality has an advantage of breed complementarity and heterosis, compared to traditionally conducted GP scheme within purebred nucleus lines [6]. Another highlight of this study is the sufficient utilization of WGS data, which was produced from cost-expensive next generation sequencing technology, to study the prediction accuracy of GP for meat quality traits in pigs. Thus, the results of our study suggested that meat quality traits can be incorporated in GP scheme and the GEBV accuracy can be improved by using WGS data. Our results also demonstrated that the genomic prediction accuracy of meat quality traits depend on marker density and GP models. However, there was no essential difference of accuracy among LD-based SNPs with different *r*^2^ cutoffs pruned. One basic assumption of GP is that each QTL affecting complex traits is in LD with at least one variant and too many SNPs or INDELs may led to biased genomic prediction for complex traits [36]. Our results showed that the prediction accuracy of LD-based SNPs with *r*^2^ < 0.2 pruned could reach the highest level of prediction ability of GBLUP in comparison with that using complete WGS data, resulting in substantially improving of computing efficiency in constructing G matrix as we point out above. In the current study, our results implied that WGS data can be utilized to explore the relationship between missing heritability and prediction accuracy of meat quality traits. Missing heritability in genome-wide association study is a major problem that limited the detection power in genomic analysis for complex traits [37]. Herein, our results showed that missing heritability may also be an important factor that limiting the prediction accuracy of GP (Fig. 4). For instance, the prediction accuracy values for meat quality traits differed between the heritability levels for a* and L*, b*, with prediction accuracies being higher for the high heritability trait (a*) than for the low heritability traits (L*, b*). The highest increase of accuracy in the analyzed meat quality traits was the up to 75% increase for b*. Furthermore, the power of genomic prediction can probably be improved by incorporating INDELs from WGS data. Our results also demonstrated that medium and high-density marker panels and variant category are beneficial for the estimation of heritability for meat quality traits.

### The superiority of MultiBLUP model that considered two random variance components over GBLUP model

In this study, we investigated the superiority of MultiBLUP model that considered different genomic information from WGS data as two random variance components over GBLUP model. Owing to the difficulty in obtaining a large-scale pig WGS dataset, the prediction accuracy of MultiBLUP incorporating INDELs from WGS data has rarely been explored. We compared the increase in prediction accuracy that can be improved by including INDELs from WGS data as additionally random variance component. The results of accuracy of MultiBLUP were increased with the increase of heritability of meat quality traits in pigs. Our results show that MultiBLUP model outperform GBLUP for all the analyzed meat quality traits when using low-density variant panels and yield a higher prediction accuracy improvement of the most 75% in b* trait. Furthermore, MultiBLUP and GBLUP models have equivalent predictive ability of meat quality traits in pigs when using medium-density and high-density SNP panels. The possible reason is that the modified genomic relationship matrix derived by the two random variance components were allowed to add higher weights to exhibit the kinship between pairs of animals [38, 39], especially for low-density panels. The advantage of MultiBLUP over GBLUP is the more the proportion of phenotypic variance explained by INDEL is larger than that explained by the remaining SNP dataset. MultiBLUP, an expansion model for GBLUP that including two random genetic effects, has an extensive use for genomic prediction in livestock animals and plant breeding. Another model that including two random genetic effects is termed as GFBLUP (genomic feature BLUP), in which a separate random genetic effect is preselected base on prior information. The performance of use MultiBLUP and GFBLUP method varied among traits. Previous studies have demonstrated that the value and superiority of GFBLUP model using prior information from WGS data, known QTLs, GWAS results, and Gene Ontology (GO) terms over GBLUP mode for reproduction traits and production traits in pigs [36], milk fatty acid composition in Holstein cows [40], body weights in broilers [41], disease resistance and growth traits in aquaculture species [42], and also for growth-related traits in crop breeding (*Arabidopsis thaliana*) [43]. However, prediction accuracy of GFBLUP differed in different scenarios [44]. The accuracy of GFBLUP may affected by the composition of genomic features. Ye et al. [45] found that GFBLUP did not yield accuracy improvement using preselected SNPs from WGS based on GWAS results, but improved the prediction accuracy with the most 60.66% increase for the starvation resistance in *Drosophila* when using preselected SNPs from eQTL (expression QTL) mapping. In this study, the MultiBLUP outperform GBLUP, at least not loss power in compared with GBLUP, for estimating the GEBV of meat quality traits. The lack of improvement in prediction accuracy of MultiBLUP using medium- and high-density variant panels may be owing to only INDELs from WGS data was used, the assignment of different weights to the ${{\varvec{G}}}_{{{\varvec{g}}}_{{\varvec{f}}}}$ and ${{\varvec{G}}}_{{{\varvec{g}}}_{{\varvec{r}}}}$ matrices did not have substantial differences [39]. Thus, it is expected that use the results of GWAS and eQTL mapping to yield higher accuracy increase of MultiBLUP and GFBLUP models, because these strategies may provide valuable insights into elucidating the genetic architecture of meat quality traits.

### Accuracy of genotype imputation and performance of GP using imputed-based WGS data

Genotype imputation is used to predict or impute the genotypes at the SNPs that are not directly genotyped in the study sample using a reference panel of haplotypes with high-density SNPs [20]. It has been proven to a cost-effective approach that greatly increase the density of genotypes to WGS level data and is widely used in the genetic studies to boost power such as in GWAS and GP. To date, several genotype imputation haplotype reference panels have been developed for animal [46], plant [47], and aquaculture species [48]. With the aid of genotype imputation, exploration of the combining admixed population into single reference population or improving the genomic prediction accuracy of combined populations is feasible and convenient in animals, such as in cattle and pig [36]. In practice, the genotyping strategy in most studies and breeding industries is to genotype animals using low-density chip, then, genotype imputation was used to improve the marker density to WGS level [10, 17, 49, 50]. However, the results of many studies demonstrated that no increase and even decrease of prediction accuracy were observed when using imputed-based WGS data in GP [12–14]. Accuracy of genotype imputation can have an effect on the prediction accuracy of GP [10]. Several factors can affect the accuracy of genotype imputation, including the genotype imputation methods used in the software [20], MAF of the imputed variants [51], and SNP array density [52]. In this study, we performed genotype imputation in the same population to acquire the accuracy of imputation from commercial medium-density marker panel (50K) to WGS level data. We found average CR to exceed 95.28% and *r*^2^ = 0.81 whereas the accuracy of genotype imputation of the used haplotypes panel is CR = 95.84% and *r*^2^ = 0.89. We observed that the average CR increased along with the number of markers in MAF bins increased. The average CR is sensitive to MAF maybe due to the count of variants with MAF higher than 0.4 in the reference panel was also small [30]. Another possible reason is that the accuracy of imputation in composite animals is related to genetic group [53, 54]. Our results showed that the accuracy of genotype imputation in crossbred populations is slightly lower than that in purebred populations [30], but it is sufficient to show the reliability of using imputed WGS data for genomic analyses. Importantly, the goal of performing genotype imputation in this study is to discard missing SNPs and INDELs in some samples and made the remained variants do not have undetected genotypes in the 1,469 sequenced pigs. From the 21,708,028 SNPs and 3,000,017 INDELs of WGS data and the 19,348,898 SNPs and 2,725,851 INDELs of imputed-based WGS data produced by genotype imputation after QC, 86.12% (18,695,907) SNPs and 70.23% (2,106,902) INDELs were present on the two sequence datasets. Thus, the efficiency of using imputed-based WGS data will be a useful strategy in GP scheme.

### Challenges for genomic prediction of meat quality traits in swine

In many cases, sequencing all animals in the reference population is not realistic owing to cost-expensive collecting of phenotypes and genotypes. However, GP of economically important traits in livestock benefits a lot from genomic data and accurately measurement of phenotypic records of animals, especially in pigs [6]. The genetic improvement of meat quality is hindered by the poorly ability to collect phenotypes from purebred pigs, which is not realistic to measure meat quality post-mortem in a large-scale purebred pig population. Then, it is expected to conduct GP scheme of meat quality in crossbred pig population. Due to the differences in genetic variants between purebred and/or crossbred pigs [55], the performance of using purebred and crossbred individuals genotypic and phenotypic data to estimate the GEBV of meat quality traits may varies in GP. The use of genotypes of crossbred pigs is non-conventional because the DLY pigs were not used in the breeding population. However, a study had shown the value of the inclusion of crossbred genomic information in the reference population when implementing GP but the prediction accuracy differed across traits [6]. Moreover, the advantage of including genotypes and phenotypes of the crossbred pigs is seemingly depending on the high correlation between purebred and crossbred individuals [56, 57]. In this respect, it is recommended to construct reference population using both purebred and crossbred individuals, but the performance of GP when selecting purebred animals in the valid population should be further explored. In this study, we sequenced all DLY pigs in reference population and conducted GP for meat quality. The inability to including purebred genomic data and phenotypic data may limit us to make deep study for further investigating of meat quality in pigs. Last but not least, the estimated heritability of meat quality in pigs is low, although full genetic variants were obtained from the inclusion of WGS data. In future research, we look forward to improve the prediction accuracies of meat quality traits by performing GWAS to map possible causal variants using WGS data and subsequently add those causal variants to a SNP panel [58]. Beyond GP of meat quality using genomic-level information, multi-omics data may contribute to a lot for improving the prediction accuracy of meat quality in swine in the future.

## Conclusions

In this study, we produced WGS data from 1,469 sequenced pigs to call variants and developed a reference panel for meat quality of GP in pigs. Results from this study showed that the accuracy of MultiBLUP model outperform GBLUP model for meat quality when using low-density variant panels. We optimized the marker density and found medium and high-density marker panels and variant category are beneficial for the estimation of heritability for meat quality traits. We conducted genotype imputation from commercial SNP panel (50K) to WGS level in the same population and found average concordance rate to exceed 95% and *r*^2^ = 0.81.

## Supplementary Information

**Additional file 1: Table S1.** Summary statistics of meat quality traits in pigs.

**Additional file 2: Fig. S1.** The relationship between the Euclidean distance calculated with meat quality phenotype values and kinship.

**Additional file 3: Table S2.** The mean accuracy and bias values of GBLUP for meat quality traits using LD pruned SNPs.

**Additional file 4: Table S3.** The estimated heritability for meat quality traits using LD pruned SNPs.

## Data Availability

The datasets used or analyzed during the present study are available from the corresponding author on reasonable request.

## Abbreviations

GBLUP: Genomic best linear unbiased prediction
GEBV: Genomic estimated breeding values
GP: Genomic prediction
GWAS: Genome-wide association study
LD: Linkage disequilibrium
QTL: Quantitative trait locus
SNP: Single nucleotide polymorphism
WGS: Whole genome sequence
