# Supplementary material for: Improving the accuracy of genomic prediction for meat quality traits using whole genome sequence data in pigs
Source: J Anim Sci Biotechnol. 2023 May 10;14:67. doi: 10.1186/s40104-023-00863-y (PMC10170792; doi:10.1186/s40104-023-00863-y)
Supplement: Supplementary file 1 — Additional file 1: Table S1. Summary statistics of meat quality traits in pigs. [file 40104_2023_863_MOESM1_ESM.docx]

**Table S1** Summary statistics of meat quality traits in pigs

| **Traits** | **N** | **Mean** | **SD** | **Min** | **Max** |
| --- | --- | --- | --- | --- | --- |
| IMF | 1469 | 1.52 | 0.64 | 0.50 | 5.00 |
| MC | 1469 | 2.19 | 0.92 | 0.50 | 6.00 |
| *L** | 1469 | 48.38 | 4.06 | 33.32 | 61.32 |
| *a** | 1469 | -0.47 | 1.09 | -5.52 | 5.22 |
| *b** | 1469 | 7.84 | 1.44 | 3.25 | 11.97 |
