# Supplementary figures and images for: Improving the accuracy of genomic prediction for meat quality traits using whole genome sequence data in pigs
Source: J Anim Sci Biotechnol. 2023 May 10;14:67. doi: 10.1186/s40104-023-00863-y (PMC10170792; doi:10.1186/s40104-023-00863-y)

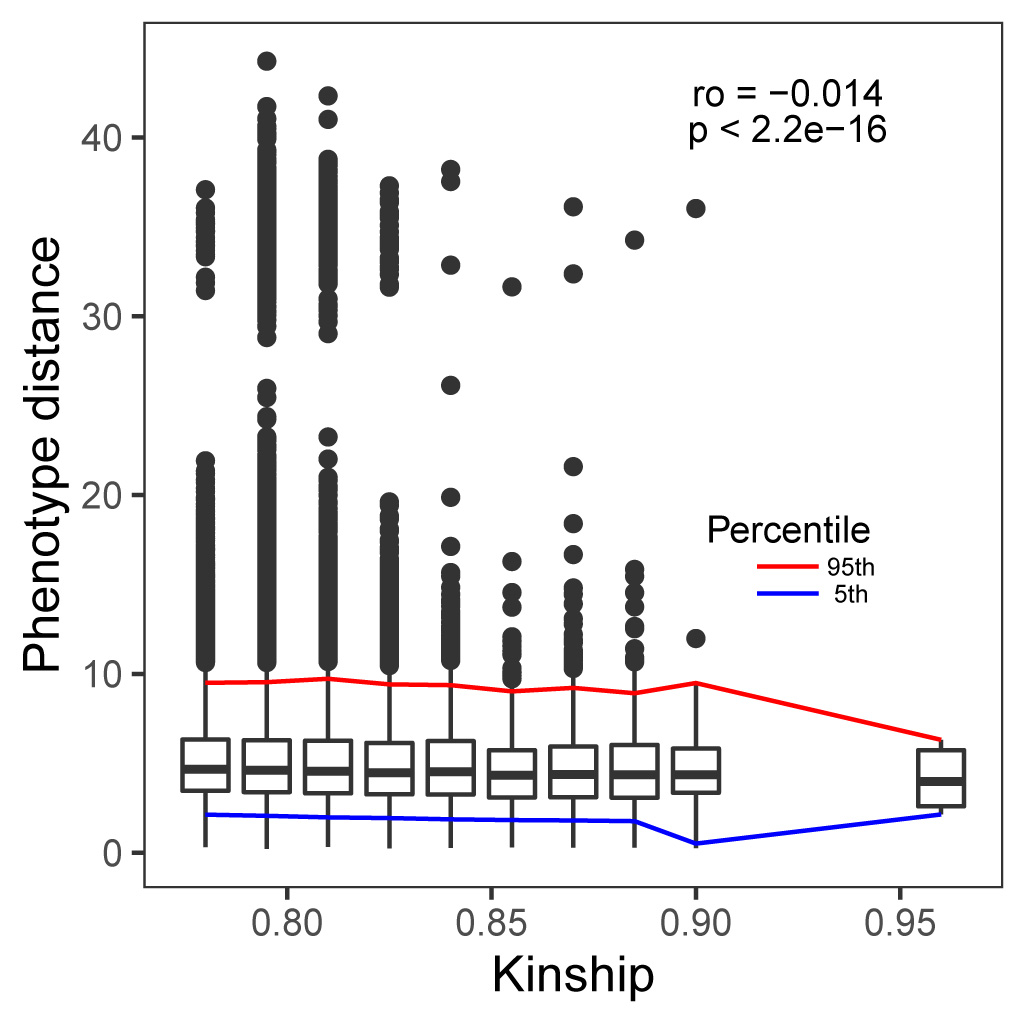

Supplement: Supplementary file 2 — Additional file 2: Fig. S1. The relationship between the Euclidean distance calculated with meat quality phenotype values and kinship. [file 40104_2023_863_MOESM2_ESM.jpg]
