# Supplementary material for: Improving the accuracy of genomic prediction for meat quality traits using whole genome sequence data in pigs
Source: J Anim Sci Biotechnol. 2023 May 10;14:67. doi: 10.1186/s40104-023-00863-y (PMC10170792; doi:10.1186/s40104-023-00863-y)
Supplement: Supplementary file 3 — Additional file 3: Table S2. The mean accuracy and bias values of GBLUP for meat quality traits using LD pruned SNPs. [file 40104_2023_863_MOESM3_ESM.docx]

**Table S2** The mean accuracy and bias values of GBLUP for meat quality traits using LD pruned SNPs

| **Model** | **LD pruned value** | **Number of SNPs** | **Traits (accuracy ± bias)** | | | | |
| --- | --- | --- | --- | --- | --- | --- | --- |
|  |  |  | **IMF** | **MC** | ***L**** | ***a**** | ***b**** |
| GBLUP | 0.2 | 1,111,198 | 0.27 ± 1.01 | 0.29 ± 1.04 | 0.17 ± 1.06 | 0.47 ± 1.00 | 0.13 ± 1.25 |
|  | 0.3 | 1,487,214 | 0.27 ± 1.01 | 0.29 ± 1.05 | 0.17 ± 1.06 | 0.47 ± 1.00 | 0.13 ± 1.25 |
|  | 0.6 | 3,216,966 | 0.27 ± 1.02 | 0.28 ± 1.05 | 0.17 ± 1.07 | 0.47 ± 1.00 | 0.13 ± 1.26 |
|  | 0.8 | 7,818,274 | 0.27 ± 1.02 | 0.28 ± 1.06 | 0.16 ± 1.07 | 0.47 ± 1.00 | 0.13 ± 1.25 |
|  | all | 18,695,907 | 0.27 ± 1.02 | 0.29 ± 1.05 | 0.16 ± 1.05 | 0.47 ± 1.00 | 0.13 ± 1.26 |
