# Supplementary material for: Improving the accuracy of genomic prediction for meat quality traits using whole genome sequence data in pigs
Source: J Anim Sci Biotechnol. 2023 May 10;14:67. doi: 10.1186/s40104-023-00863-y (PMC10170792; doi:10.1186/s40104-023-00863-y)
Supplement: Supplementary file 4 — Additional file 4: Table S3. The estimated heritability for meat quality traits using LD pruned SNPs. [file 40104_2023_863_MOESM4_ESM.docx]

**Table S3** The estimated heritability for meat quality traits using LD pruned SNPs

| **LD pruned value** | **Traits (Mean ± SE)** | | | | |
| --- | --- | --- | --- | --- | --- |
|  | **IMF** | **MC** | ***L**** | ***a**** | ***b**** |
| 0.2 | 0.28 ± 0.05 | 0.32 ± 0.05 | 0.17 ± 0.05 | 0.64 ± 0.05 | 0.12 ± 0.04 |
| 0.3 | 0.28 ± 0.05 | 0.32 ± 0.05 | 0.18 ± 0.05 | 0.64 ± 0.05 | 0.12 ± 0.04 |
| 0.6 | 0.29 ± 0.05 | 0.33 ± 0.05 | 0.18 ± 0.05 | 0.67 ± 0.05 | 0.13 ± 0.04 |
| 0.8 | 0.28 ± 0.05 | 0.32 ± 0.05 | 0.17 ± 0.05 | 0.65 ± 0.05 | 0.12 ± 0.04 |
| All | 0.26 ± 0.05 | 0.30 ± 0.05 | 0.15 ± 0.04 | 0.61 ± 0.05 | 0.11 ± 0.04 |
